# Immune-Triggered Forms of Plasticity Across Brain Regions

**DOI:** 10.3389/fncel.2022.925493

**Published:** 2022-07-22

**Authors:** Momoka Hikosaka, Takeo Kawano, Yayoi Wada, Tomoki Maeda, Takeshi Sakurai, Gen Ohtsuki

**Affiliations:** Department of Drug Discovery Medicine, Graduate School of Medicine, Kyoto University, Kyoto, Japan

**Keywords:** immune-triggered plasticity, brain immune cells, inflammatory mediators, synaptic transmission, intrinsic excitability, cerebellum, medial PFC (mPFC), psychiatric diseases

## Abstract

Immune cells play numerous roles in the host defense against the invasion of microorganisms and pathogens, which induces the release of inflammatory mediators (e.g., cytokines and chemokines). In the CNS, microglia is the major resident immune cell. Recent efforts have revealed the diversity of the cell types and the heterogeneity of their functions. The refinement of the synapse structure was a hallmark feature of the microglia, while they are also involved in the myelination and capillary dynamics. Another promising feature is the modulation of the synaptic transmission as synaptic plasticity and the intrinsic excitability of neurons as non-synaptic plasticity. Those modulations of physiological properties of neurons are considered induced by both transient and chronic exposures to inflammatory mediators, which cause behavioral disorders seen in mental illness. It is plausible for astrocytes and pericytes other than microglia and macrophage to induce the immune-triggered plasticity of neurons. However, current understanding has yet achieved to unveil what inflammatory mediators from what immune cells or glia induce a form of plasticity modulating pre-, post-synaptic functions and intrinsic excitability of neurons. It is still unclear what ion channels and intracellular signaling of what types of neurons in which brain regions of the CNS are involved. In this review, we introduce the ubiquitous modulation of the synaptic efficacy and the intrinsic excitability across the brain by immune cells and related inflammatory cytokines with the mechanism for induction. Specifically, we compare neuro-modulation mechanisms by microglia of the intrinsic excitability of cerebellar Purkinje neurons with cerebral pyramidal neurons, stressing the inverted directionality of the plasticity. We also discuss the suppression and augmentation of the extent of plasticity by inflammatory mediators, as the meta-plasticity by immunity. Lastly, we sum up forms of immune-triggered plasticity in the different brain regions with disease relevance. Together, brain immunity influences our cognition, sense, memory, and behavior *via* immune-triggered plasticity.

## Introduction

Brain immunity is the defense system for the invasion of microorganisms and viruses. Such immune system to protect our body from diseases and infections is mainly ascribed to the functions of immune cells: microglia, macrophage, myeloid cells, granulocytes, dendritic cells, natural killer cells, T cells, and B cells (Medzhitov and Janeway, 2000; Strecker et al., 2017). Aberrance of the brain immune system is one of the promising features of psychiatric disorders as well as neurodegenerative diseases (Yolken and Torrey, 1995; Gruol and Nelson, 1997; Parker-Athill and Tan, 2010; Khandaker et al., 2015; Colonna and Butovsky, 2017; Nimgaonkar et al., 2017; Barichello et al., 2019; Pape et al., 2019; Ozaki et al., 2021; Segawa et al., 2021). The immune system of mammals, including humans, is divided into adaptive immunity and innate immunity. Adaptive immunity (i.e., acquired immunity) is acquired in response to the stimulation of foreign substances. And it is the specific antibodies and T cell receptors that recognize foreign substances through antigen presentation. Immunological memory is an important characteristic of adaptive immunity, and this acquired system enables us to respond instantly to the identical antigen that had invaded our body previously. On the other hand, innate immunity is another dominant immune system, which recognizes foreign substances against targeting molecular patterns peculiar to microorganisms. The pattern recognition molecules are the complements, lectins, Toll-like receptors, etc., which are encoded in the genome (Hoshino et al., 1999; Pandey et al., 2014). Innate immunity does not have a complex and specific immune memory, like adaptive immunity, and responds immediately to specific components of pathogens. However, it could be considered that there is a different form of the memory system in innate immunity and distinct microglia/macrophage responses, as seen in the disease-associated microglia (DAM), which are thought as the microglia population that acquired pro-inflammatory activity by altering the expression of genes involved in the lysosomal/phagocytic pathway, endocytosis and the regulation of immune responses in response to central nervous system (CNS) pathologies (Deczkowska et al., 2018). Whereas disorders of acquired immunity are common in autoimmune diseases, innate immune system anomalies are often noted in many psychiatric disorders. At the evolutionary origin, acquired immunity appears in vertebrate lampreys and hagfishes (*Cyclostomatas*). On the other hand, innate immunity existed long before the advent of vertebrates. The complement system has been found in ascidians and sea urchins, and Toll-like receptors are already present in cnidarians (Nonaka and Yoshizaki, 2004; Brennan and Gilmore, 2018; Liu et al., 2020).

Inflammatory mediators, such as cytokines and chemokines, are molecules released from immune cells in response to the damage-associated molecular patterns (DAMPs), pathogen-associated molecular patterns (PAMPs), and neurodegeneration-associated molecular patterns (NAMPs), and the mediators play distinct roles in both physiology and pathology in the CNS (Hopkins and Rothwell, 1995; Gruol and Nelson, 1997; Deczkowska et al., 2018). Regarding disease association with the brain immunity, excessive inflammatory cytokines, disruption of brain vasculature systems, the proliferation of activated immune cells in the parenchyma, and malformation of neurons are observed in the various animal models emerging the symptoms of psychiatric disorders (Hopkins and Rothwell, 1995; Gilmore et al., 2004; Choi et al., 2016; Menard et al., 2017; Greene et al., 2018; Nie et al., 2018). Previously, researchers had regarded the cerebellum as not an important brain site in the development of stress responses by aberrant immunity (Nguyen et al., 1998). The current view suggests that the cerebellum is vulnerable to aberrant immunity and infection in the early developmental period, which is the new region causing the emergence of psychiatric diseases including autism spectrum disorders, mood disorder, and schizophrenia (Schmahmann, 2004; Tsai et al., 2012, 2018; Piochon et al., 2014; Moberget et al., 2018; Yamamoto et al., 2019; Cook et al., 2021; Ozaki et al., 2021; Baek et al., 2022; Hwang et al., 2022). Typically, in autism spectrum disorder (ASD)-patient brains, the cerebellum presented various pathological impairments, including the uneven structure of the Purkinje cell layer and granular cell layer, marked activation of microglia and reactive Bergmann's astroglia, and accumulation of perivascular macrophages and microglia. The cytokine profiling also indicated that monocyte chemoattractant protein 1 [MCP-1; known as CCL2: chemokine (C-C motif) ligand 2], interleukin-6 (IL-6), and transforming growth factor-β1 (TGFβ1) were the most prevalent cytokines in ASD brain tissues. Cerebrospinal fluid from patients showed a unique pro-inflammatory profile of cytokines, including a marked increase in interferon-γ (IFN γ), TGFβ, MCP-1, IL-8, fibroblast growth factor (FGF)-9, and vascular endothelial growth factor (VEGF) (Vargas et al., 2005; Parker-Athill and Tan, 2010). In maternal immune activation (MIA) mice by administration of Poly(I:C) to the pregnant mothers, adult offspring show ASD-like behaviors and a deficit of Purkinje cells in the middle lobes (e.g., lobule VIa-VII) of the cerebellum (Shi et al., 2009; Naviaux et al., 2013), presumably reflecting the vulnerability of the vasculature and promoted access of the peripheral immunity in the developmental period. A spatially localized delay of granule-cell migration is also observed during the cerebellar development in the MIA exposed animals (Shi et al., 2009). Thus, the cerebellum is expected to be one of the brain regions implicated in developmental disorders with distinct neuroinflammation. Despite that, the mechanisms of how inflammatory mediators modulate neuronal activity as the synaptic transmission and the intrinsic excitability are not utterly elucidated. We speculate that the cerebellum is vulnerable to immune-cell invasion through the fourth ventricle, capillaries, and pial vasculatures between folia. For a better understanding of cerebellar inflammation and its relevance to psychiatric disorders, an overview of recent studies regarding immune-triggered plasticity across the brain is required. Such effort should help advance this field and find therapeutic targets.

## Microglia Heterogeneity and Roles for Modulating Activity of Neurons

The resident immune cells in the brain, microglia, are the most abundant immune cells in the brain. Microglia originate from erythromyeloid progenitors in the yolk sac and invade the brain before forming the blood-brain barrier (BBB) (Ling and Wong, 1993; Nakajima and Kohsaka, 1993; Ginhoux et al., 2010; Ginhoux and Prinz, 2015). Microglia account for 5–12% of the total number of cells present in the mouse brain (Lawson et al., 1990) and 0.5–16.6% of the total number of cells in the human brain (Mittelbronn et al., 2001), depending on the brain region. Microglial cell densities across mammalian species remain conserved (e.g., around 6% of all cells in the mammalian cortex) for over 200 million years (Dos Santos et al., 2020). Askew et al. (2017) demonstrated that the number of microglial cells remains steady from early postnatal stages until aging and is maintained by the regulated coupling of proliferation and apoptosis without a contribution from circulating progenitors (Askew et al., 2017).

Classically, microglia could be divided into two phenotypes: amoeboid microglia and ramified microglia (Ling and Wong, 1993). In the adult brain, resting-state microglia are dynamic and have many functions from the homeostasis of the brain-parenchymal milieu to the refinement of the neural circuit (Nimmerjahn et al., 2005). It is well-known that clearance of the protein aggregates, damaged organelles, redundant synapses, and dead cells are crucial for the maintenance of brain health and brain development (Colonna and Butovsky, 2017; Deczkowska et al., 2018). When microglia are reactivated *via* the innate immune system by DAMPs and PAMPs, they transform into activated microglia which change gene expression patterns and their morphology. They have the characteristic of the high ability of proliferation, migratory, and phagocytosis, and release various inflammatory mediators. Recent studies indicate microglia are heterogeneous across brain regions in terms of morphology, signaling pathways, and gene expression patterns (Grabert et al., 2016; Ayata et al., 2018; Silvin and Ginhoux, 2018; Süß et al., 2020). The phenotypes as transcripts and morphology of microglia differ from the localized region or age (De Biase et al., 2017; Masuda et al., 2019). Ablation and repopulation of microglia also alter phenotypes and localizations (De Biase et al., 2017; Masuda et al., 2019). Our current understanding of gene expression patterns is based on many transcriptome studies and morphological analyses. While we admit the importance of the transcriptome, such studies do not provide any information on functional protein expression. Upcoming multiplexed proteome studies, such as imaging masscytometry, should overcome the points (Fernández-Zapata et al., 2020). As abovementioned, there is no complex and specific immune memory in innate immunity, but the feature of heterogeneity might be a form of the memory in innate immunity.

Importantly, microglia are one of the major resources of inflammatory cytokine releases in response to peripheral inflammation, invaded microbes, bacterial endotoxin, and nucleic acids. For instance, transient exposure of brain slices to the Gram-negative bacterial endotoxin, lipopolysaccharide (LPS), activates microglia through Toll-like receptor 4 (TLR4), a pattern recognition receptor. Exposure to LPS facilitates the vesicular release at hippocampal excitatory presynaptic terminals (Pascual et al., 2012). It was also shown that under the hypoxia condition exposure to LPS could depress the post-synaptic efficacy of glutamate receptors by the superoxide and nitric oxide production (Zhang et al., 2014). The number of studies on such immune-triggered modulations of synaptic transmission in the CNS is increasing these days (Pascual et al., 2012; Parkhurst et al., 2013; Zhan et al., 2014; Zhang et al., 2014; Yamamoto et al., 2019; Di Filippo et al., 2021). Therefore, brain immunity is the triggering factor of synaptic plasticity *via* immune receptors, such as pattern-recognition receptors and cytokine receptors, expressed in the neurons. In contrast, activated microglia also modulate the non-synaptic membrane excitability of neurons in the neocortex, hippocampus, amygdala, and cerebellum (Gao et al., 2014; Klapal et al., 2016; Tzour et al., 2017; Duan et al., 2018; Luo et al., 2019; Yamamoto et al., 2019; Zheng et al., 2021; Yamawaki et al., 2022). In a study by Zhang et al. (2014), changes in waveforms of field EPSPs after microglia activation by exposure to LPS were shown, and the result not only indicated the long-term depression (LTD) of the synaptic transmission but also implied the modulation of the late component of evoked synaptic transmission as an ion-channel modulation. Nevertheless, the cellular mechanism for inducing the long-term plasticity of intrinsic excitability is known in a limited number of cell types. The neuromodulation of the microglial functions is not only for the synaptic plasticity (i.e., synaptic efficacy and synaptic structure) but also for the excitability of neurons and even their dendrites (Tables 1, 2). And the involved immune cell types including microglia and the released inflammatory mediators are just started to be identified.

**Table 1:** Immune-triggered modulation of the synaptic transmission of neurons.

**Brain region**	**Species**	**Immune stimulator**	**Triggering cell type**	**Inflammatory cytokines**	**Neuron types**	**Synapse types**	**Pre/post**	**Plasticity**	**References**
Cerebellum	Balb/c mice (P8-week)	GROα (<5 min)	Not identified	GROα (CXCL1), IL-8 (CXCL8 )	Purkinje cell	Glutamate	Pre/post	PSC: Freq↑; eEPSC: Amp↑; LTD suppression	Giovannelli et al., 1998
Cerebellum	ACI/T rats (P4–8 weeks)	GROβ (<4 min)	Not identified	GROβ (CXCL2)	Purkinje cell	Glutamate	Pre	eEPSC: Amp ↑; mPSC: Freq↑	Ragozzino et al., 1998
Cerebellum	C57Bl/6J mice (male, P2 months), Sprague-Dawley rats (male, P21–32 days)	LPS (<10 min)	Microglia	TNFα	Purkinje cell	Glutamate	Pre/post	s/mEPSC: Freq↑, Amp↑	Yamamoto et al., 2019
Hippocampus	C57Bl/6J mice (P1–3 months), Sprague-Dawley rats (P18–23 days)	LPS + low O_2_	Microglia	Superoxide	CA1 pyramidal cell	Glutamate	Post	fEPSP: Slope↓ (AMPAR- LTD); mEPSC: Amp↓, Freq no change	Zhang et al., 2014
Hippocampus	C57Bl/6J mice (P15–21 days)	LPS	Microglia	ATP->P2Y1R	CA1 neurons	AMPA	Pre	sEPSC: Freq↑	Pascual et al., 2012
Hippocampus	Sprague-Dawley rats (P2–4 weeks)	TNFα (2–3 h)	Not identified	TNFα	CA1 pyramidal cell	AMPA	Post	mEPSC: Amp↑, Freq no change	Stellwagen et al., 2005
Hippocampus	C57Bl/6J mice (P5–8-week)	TNFα	Not identified	TNFα	CA1 pyramidal cell	GABA	Post	mIPSC: Amp↓	Pribiag and Stellwagen, 2013
Hippocampus	C57Bl/6 mice (female, P6–8 weeks)	IL-1β (30 ng/ml for 10 min)	Microglia	IL-1β	CA1 pyramidal cell	GABA	Pre/post	sIPSC: Amp↓, Freq↓	Nisticò et al., 2013
Hippocampus	Sprague-Dawley rats (P14–22 days), C57Bl6 mice	CX3CL1 (100 nM for 10 min)	Not identified	CX3CL1	CA1 pyramidal cell	AMPA (Schaffer collaterals)	Post	eEPSC: Amp ↓; PPR: no change	Ragozzino et al., 2006
Hippocampus	Sprague-Dawley rats (P14–17 days)	LPS (30 min)	Microglia	TNFα?, IL-1β?	CA1 pyramidal cell	Glutamate	Not identified	eEPSC: Amp↑; eIPSC: no change	Gao et al., 2014
Hippocampus	Sprague-Dawley rats (male, P15–30 days)	CCL2 (<5 min)	Not identified	CCL2	CA1 region, Schaffer collateral	Glutamate	Pre	eEPSC: Amp↑; sEPSC: Freq↑	Zhou et al., 2011
Hippocampus	hGFAP-CCL2 mice (SJL background, P7–12 months)	CCL2 (constitutive expression in astrocytes)	Astrocytes	CCL2	CA1 region, Schaffer collateral	Glutamate	Pre	Presynaptic volley↓ fEPSP↓; PPR↑ (fEPSP and Pop spike); PTP&STP of fEPSP↑	Nelson et al., 2011
Hippocampus and cortical S1 areas	C57Bl/6 mice (P14 days or P50-P60 days)	LPS, Poly(I:C)	Vascular pericytes	CCL2/MCP-1	Pyramidal cell, and DG granule cell	AMPA	Post	mEPSC: Freq↑, Amp no change	Duan et al., 2018
Hippocampal dentate gyrus	C57Bl/6 mice (P17–25 days, and P60–90 days)	TNFα	Astrocytes	TNFα	Granule cell	Glutamate	Pre	mEPSC: Freq↑, Amp no change	Habbas et al., 2015
Hippocampus	C57Bl/6J mice (P19–21-day)	LPS (100 ng/mL for 1 h)	Microglia	ATP	CA3 pyramidal cell	Glutamate (mossy fiber synapses)	Pre	eEPSC: PPR↓, Facilitation↓	George et al., 2016
Hippocampal culture	Rat culture	TNFα	Glia (astrocyte)	TNFα	Cultured neurons in mixed neuronal-glial culture	AMPA	Post	Surface expression (AMPAR)↑; mEPSC↑	Beattie et al., 2002
Hippocampal neuronal culture	Wistar rat culture	IL-1β (>10 ng/ml for 2-5 min)	Not identified	IL-1β	Cultured neurons	NMDA, L-Ca^2+^ channel	Post	s/mEPSC: freq↓; NMDA evoked current: Amp↑; VDCC (mainly L type)↑	Yang et al., 2005
Hippocampal primary culture	Rats	IL-1β (0.025-0.100 ng/ml for 1.5 min)	Not identified	IL-1β	Cultured neurons	NMDA	Post	[Ca2+]i↑	Viviani et al., 2003
Neocortex	Wistar rats (male, P10–84 days)	IFN-γ (1000 IU/ml)	Not identified	IFN-γ	L5 pyramidal cell	GABA	Pre	eIPSC: Amp↑; s/mIPSCs: Amp↑, Freq no change	Janach et al., 2020
Medial prefrontal cortex	Sprague-Dawley rats (P21–32 days old)	LPS (<10 min)	Microglia	TNFα?	L5 pyramidal cell	GABA	Pre	sEPSC: Freq no change, Amp no change; sIPSC: Freq↓, Amp no change; mIPSC: Freq no change, Amp no change	Yamawaki et al., 2022
Medial prefrontal cortex	C57Bl/6J mice (P1–2-month, both sex)	LPS (i.p., 2 h)	Microglia	LPS	L2/3 pyramidal cell	GABA Ra5	Pre	mEPSC: Freq no change, Amp no change; mIPSC: Freq↓, Amp↓	Jiang et al., 2022
Temporal cortex	Rats	LPS (i.p.)	Not identified	IL-6 (10 ng/mL)	L2/3 pyramidal cell	GABA	Post	eEPSC: Amp no change; eIPSC Amp↓, PPR no change	Garcia-Oscos et al., 2012
Dorsal striatum	C57Bl/6J mice (male, P8–11 weeks)	TNFα (1–2 h)	Not identified	TNFα	GABAergic medium spiny neuron	AMPA	Post	sEPSC: Amp↓	Lewitus et al., 2014
Basolateral amygdala	C57Bl/6 mice (male, P9–10 weeks)	LPS (24 h after i.p.)	Microglia	TNFα?, IL-1β?	Glutamatergic projection neurons	AMPA	Pre	mEPSC: Freq↑, Amp no change	Zheng et al., 2021
		TNFα (2–3 h)	Not identified	TNFα		GABA_A_	Post	mIPSC: Amp↓, Freq no change	
Nucleus accumbens	C57Bl/6J mice (male, P8–12 weeks)	TNFα	Microglia	TNFα	NAc core medium spiny neuron	AMPA	Post	eEPSC: Amp↓	Lewitus et al., 2016
Lateral habenula	C57Bl/6J mice (P4–10 weeks)	Morphine withdrawal	Microglia?	TNFα	LH neurons	Glutamate	Post	eEPSC(AMPA): Amp↓	Valentinova et al., 2019
Ventrolateral periaqueductal gray (vlPAG)	vGAT-L10A-GFP mouse (C57BL6/J background, female, P2-5 months), TH-eGFP	TNFα (100 ng/ml for 1 h)	Not identified	TNFα	GABA neurons	Glutamate	Pre/Post	sEPSC: Freq↓, Amp↓; sIPSC: Freq no change, Amp no change	Pati and Kash, 2021
	mouse (Swiss Webster background, female, P2-5 months)				DA neurons	Glutamate	Pre	sEPSC: Freq↓, Amp no change; sIPSC: Freq no change, Amp no change	
Spinal cord	Sprague-Dawley rats (P7–14 and P26–30 days)	PGE2, 10-min application	Not identified	PGE2	Dorsal horn lamina II neurons	Glycine	Post	Glycinergic eIPSC: Amp↓; GABAA-eIPSC no change; AMPA-eEPSC no change; NMDA-eEPSC no change; mIPSC: Amp↓, Freq no change	Ahmadi et al., 2002
Spinal cords (thoracolumbar spine (T11–L3) )	C57Bl/6 mice (both sexes, P21–37 days)	LPS, 30-min application	Microglia?	PGE2/EP2R	Substantia gelatinosa neurons	Glycine	Post	Glycinergic PSC: Amp↓, GABAergic PSC: no change	Cantaut-Belarif et al., 2017
Spinal cord	C57Bl/6J mice (P4–6 weeks)	CCL2 (<1 min )	Not identified	CCL2	Dorsal horn lamina II neurons	NMDA	Pre	Glutamate release↑	Ma et al., 2020
L4–L5 lumbar spinal cord segment	Wistar rats (female, P2–3 -month and P2-3-week)	Bone Cancer Pain; IL-18 (10 ng/ml) for 2 min	Microglia?	IL-18	Superficial dorsal horn neurons	Glutamate	Pre	mEPSC: Freq↑, Amp no change	Yang et al., 2015
L4–L5 lumbar spinal cord	C57Bl/6J mice (male, P8–10 weeks)	IL-17 (<1 min)	Spinal astrocytes	IL-17	Lamina IIo SOM+ neurons	AMPA, NMDA, Pre GABA	Pre	sEPSC: Freq↑, Amp no change sIPSC: Freq↓, Amp↓	Luo et al., 2019
L4–L5 lumbar spinal cord	Adult rats		Not identified	IL-1β	Substantia gelatinosa neurons	AMPA, NMDA, GABA, Glycine	Not identified	sEPSC: Freq↑, Amp↑; sIPSC: Freq↓, Amp↓	Kawasaki et al., 2008
				TNFα				sEPSC: Freq↑, Amp no change	
				IL-6				sEPSC: Freq no change, Amp no change; sIPSC: Freq↓, Amp no change	
L4-L5 lumbar spinal cord	FVB/NJ mice (P4–6 weeks), CB6-Tg(Gad1-EGFP)G42Zjh/J mice	TNFα (10 & 50 ng/ml, 2 min)	Not identified	TNFα/TNFR1	Substantia gelatinosa neurons	GABA, Glycine	Pre	sEPSC: Freq↑, Amp no change; sIPSCs: Freq↓, Amp no change; mIPSC: freq↓, Amp no change; spontaneous activity: Freq↓; Ih current↓	Zhang et al., 2010
Spinal cord (L1-S3)	Sprague-Dawley rats (P5–6 weeks)	t-BOOH (: ROS s donor, 10 mM, 5 min)	Not identified	ROS	Substantia gelatinosa neurons	Glutamate	Pre	sEPSCs: Freq↑, Amp↑; slow inward current produced; mEPSCs: Freq↑, Amp↑	Nishio et al., 2013
Spinal cord culture	Wistar rats	MCP-1/CCL2 (3, 10, 30, 50 nM; co-application of GABA 100 μM)	Not identified	CCL2	Cultured neurons	GABA	Post	GABA-induced current: Amp↓	Gosselin et al., 2005

*We show a summary table of the immune-triggered modulation of the synaptic efficacy of neurons in different brain regions. We categorized referenced studies by brain region, animal species, stimulants for the immune cell, immune-cell or glia type, inflammatory mediators, neuron types, synapse types, pre-/post- synaptic location, and forms of plasticity (i.e., LTP and LTD). The outcomes are indicated by different signs: increased (↑), reduced (↓), and unchanged. Paired-pulse ratio (PPR) is defined as the ratio of response against 2nd stimulation to response against the initial one after a conditioning or reagent administration. Thus, the PPR indicates whether the presynaptic release probability of the synaptic vesicles is changed by the plasticity induction (Blitz et al., 2004)*.

*Amp, amplitude; eEPSC, evoked excitatory postsynaptic current; eIPSC, evoked inhibitory postsynaptic current fEPSP, field excitatory postsynaptic potential; Freq, frequency; mEPSC, miniature EPSC; mIPSC, miniature IPSC; mPSC, miniature postsynaptic current; PPR, paired-pulse ratio; PSC, postsynaptic current; PTP, post-tetanic potentiation; sEPSC, spontaneous EPSC; sIPSC, spontaneous IPSC; STP, short-term synaptic plasticity; VDCC, voltage-dependent Ca^2+^-channel*.

**Table 2:** Immune-triggered modulation of the intrinsic excitability of neurons.

**Brain region**	**Species**	**Immune stimulator**	**Triggering cell type**	**Inflammatory cytokines**	**Neuron types**	**Types of excitability change**	**Ion-channels**	**Plasticity**	**References**
Cerebellum	GFAP-IL-6 mice (P31–73 days)	IL-6 (constitutive expression In astrocytes)	Astrocytes (Bergmann glia)	IL-6	Purkinje cell	FF↓	Not identified	Excitability↓, oscillatory firing patterns↑, climbing fire response ↑	Nelson et al., 1999
Cerebellum	Rats (P179625 days), C57BL/6 mice (male, P6 weeks)	TNFα (100 ng/ml for 40 min)	Astrocytes (Bergmann glia)	TNFα	Purkinje cell	FF↑	Ih (HCN)↓	Excitability↑	Shim et al., 2018
Cerebellum	C57Bl/6J mice (male, P2 months), Sprague-Dawley rats (male, P21–32 days)	LPS (<10 min)	Microglia	TNFα	Purkinje cell	FF↑, Dendrite excitability↑	SK2↓	Excitability↑, IE-LTP	Yamamoto et al., 2019
Cerebellar culture	Sprague-Dawley rats	IL-6 (10 ng/ml, 14 days)	Not identified	IL-6	Cultured Purkinje cells	FF↓	Not identified	Excitability↓	Nelson et al., 2002
		IL-6 (1 ng/ml, 21 days)	Not identified	IL-6		FF↑	Not identified	Excitability↑	
Cerebellar culture	Sprague-Dawley rats	IL-6 (5 ng/ml, 10 days)	Not identified	IL-6	Cultured Purkinje cells	Ca^2+^ responses↑	Not identified	DHPG- and K^+^-evoked intracellular Ca^2+^↑	Nelson et al., 2004
Hippocampus	Sprague-Dawley rats (male, adult)	TNBS-induced colitis	Microglia	TNFα	CA1 pyramidal neuron	FF↑	Not identified	Excitability↑	Riazi et al., 2008
Hippocampus	Sprague-Dawley rats (male, P15–30 days)	CCL2 (<5 min)	Not identified	CCL2	CA1 pyramidal neuron	FF↑	Not identified	Excitability↑, Resting Vm depolarized	Zhou et al., 2011
Hippocampus	hGFAP-CCL2 mice (SJL background, P7-12 months old)	CCL2 (constitutive expression in astrocytes)	Astrocytes	CCL2	Schaffer collateral-CA1 region (extracellular recording)	FF↓	Not identified	Population spike↓	Nelson et al., 2011
Hippocampus	C57Bl/6J mice (P15–21 days)	LPS (<10 min)	Microglia	?	CA1 neurons	FF↑ (epileptiform bursting, 0 mM Mg)		Excitability↑	Pascual et al., 2012
Hippocampal culture, microglia-included	Wistar-Hannover rat culture	TNFα (4 days), IL-18 (4 days)	Microglia	TNFα, IL-18	Bipolar cell, and pyramid shaped cell	Na^+^ current density↑	Voltage-gated sodium channels	Na^+^ current density↑	Klapal et al., 2016
Hippocampus	Sprague-Dawley rats (P14–17 days)	LPS (30 min)	Microglia	TNFα? IL-1β?	CA1 pyramidal neuron	FF↑, Rheobase↓	Not identified	Excitability↑	Gao et al., 2014
Hippocampus	Sprague-Dawley rats (male, adult)	LPS	Microglia	ATP (astrocyte)	CA1 pyramidal neuron	FF↑, Rheobase↓	Not identified	Excitability↑	Tzour et al., 2017
Hippocampus and cortical S1 areas	C57BL/6J background (male and female, P14 days or P50-60 days)	LPS, Poly(I:C)	Vascular pericytes	CCL2/MCP-1	Pyramidal cell, and granule cell	FF↑, Rinput↑, Threshold↓	Not identified	Excitability↑	Duan et al., 2018
Medial prefrontal cortex	Sprague-Dawley rats (male, P21–32 days)	LPS (<10 min)	Microglia	TNFα	L5 and L2/3 pyramidal cell	FF↓	SK1↑	Excitability↓	Yamawaki et al., 2022
Visual cortex	Rat pups (P4–6 days)	BDNF (20 ng/ml. 2 days with TTX)	Not identified	BDNF	Cortical pyramidal neuron culture	FF↓, Rheobase↑, Threshold↓	Not identified	Excitability↓ (Against TTX induced Homeostatic plasticity)	Desai et al., 1999
Basolateral amygdala	C57Bl/6 mice (male, P9–10 weeks)	LPS (24 h after i.p.)	Microglia	TNFα?, IL-1β?	Glutamatergic projection neurons	FF↑, AHP↓	SK2↓	Excitability↑	Zheng et al., 2021
Ventrolateral periaqueductal gray	vGAT-L10A-GFP mice (C57BL6/J background, female, P2–5 months)	TNFα (100 ng/ml for 1 h)	Not identified	TNFα	GABA neurons	FF↑, Rheobase↓	Not identified	Excitability↑	Pati and Kash, 2021
					DA neurons	FF↓, Rheobase↑	Not identified	Excitability↓	
Spinal cord	Sprague-Dawley rats (male, >P50 days	ATP (50 μM, 1 h)	Microglia	BDNF	Dorsal horn spinal lamina I (LI) neurons	GABA response changes	Not identified	Anion reversal potential depolarization	Coull et al., 2005
Spinal cord	C57Bl/6 mice (both sexes, P5–7 weeks), Human DRGs (L4–L5)	CCI, PD-1	Not identified	CCI, PD-1	DRG neurons	FF↑	Not identified	Excitability↑, INa↓	Chen et al., 2017
L4-L5 lumbar spinal cord	FVB/NJ mice (P4-6 weeks old), CB6-Tg (Gad1-EGFP) G42Zjh/J mice	TNFα (10 & 50 ng/mg, 2 min)	Not identified	TNFα/TNFR1	Substantia gelatinosa GABAergic neurons in spinal cord dorsal horn	FF↓	Ih (HCN)↓	Excitability↓, Ih current↓	Zhang et al., 2010
L4-L5 lumbar spinal cord	C57BL/6J mice (male, P8–10 weeks)	IL-17 (10 ng/mL, 2–5 min)	Spinal astrocytes	IL-17	DRG small neurons	FF↑, Vm↑, Rheobase↓	Not identified	Excitability↑	Luo et al., 2019
Dorsal root ganglion (DRG)	Sprague-Dawley rats (male, P6-9- week)	TNFα	Not identified	TNFα	DRG neuron culture	FF↑, Threshold↓	TTX-resistant Na channels↑: Nav 1.8 and 1.9	Excitability↑	Gudes et al., 2015

*We tabulated a summary of the immune-triggered modulation of the intrinsic excitability of neurons in different brain regions. We categorized studies by brain region, animal species, stimulants for the immune cell, immune-cell or glia type, inflammatory mediators, neuron types, types of excitability changes, modulated ion-channels, and forms of plasticity of intrinsic excitability. The outcomes are indicated by different signs: increased (↑), reduced (↓), and unchanged*.

*AHP, after hyperpolarization; FF, firing frequency; Rinput, input resistance; Vm, membrane voltage*.

Considering microglia as the most abundant immune cells in the brain parenchyma, they should play the most responsible roles for brain homeostasis and disease state. For the induction of immune-triggered plasticity, however, the mechanisms for induction are utterly the frontier in the present neuroscience field. In the following sections, we review recent advances in our understanding of the synaptic and non-synaptic plasticity induction by microglia and other immune cells, their contribution to the homeostasis of network dynamics throughout the CNS regions, and their relevance to the emergence of psychiatric diseases. We also highlight the ion channels and molecular signaling implicating the immune-triggered plasticity.

## Immune-Triggered Plasticity in the Cerebellum

**Figure 1:**
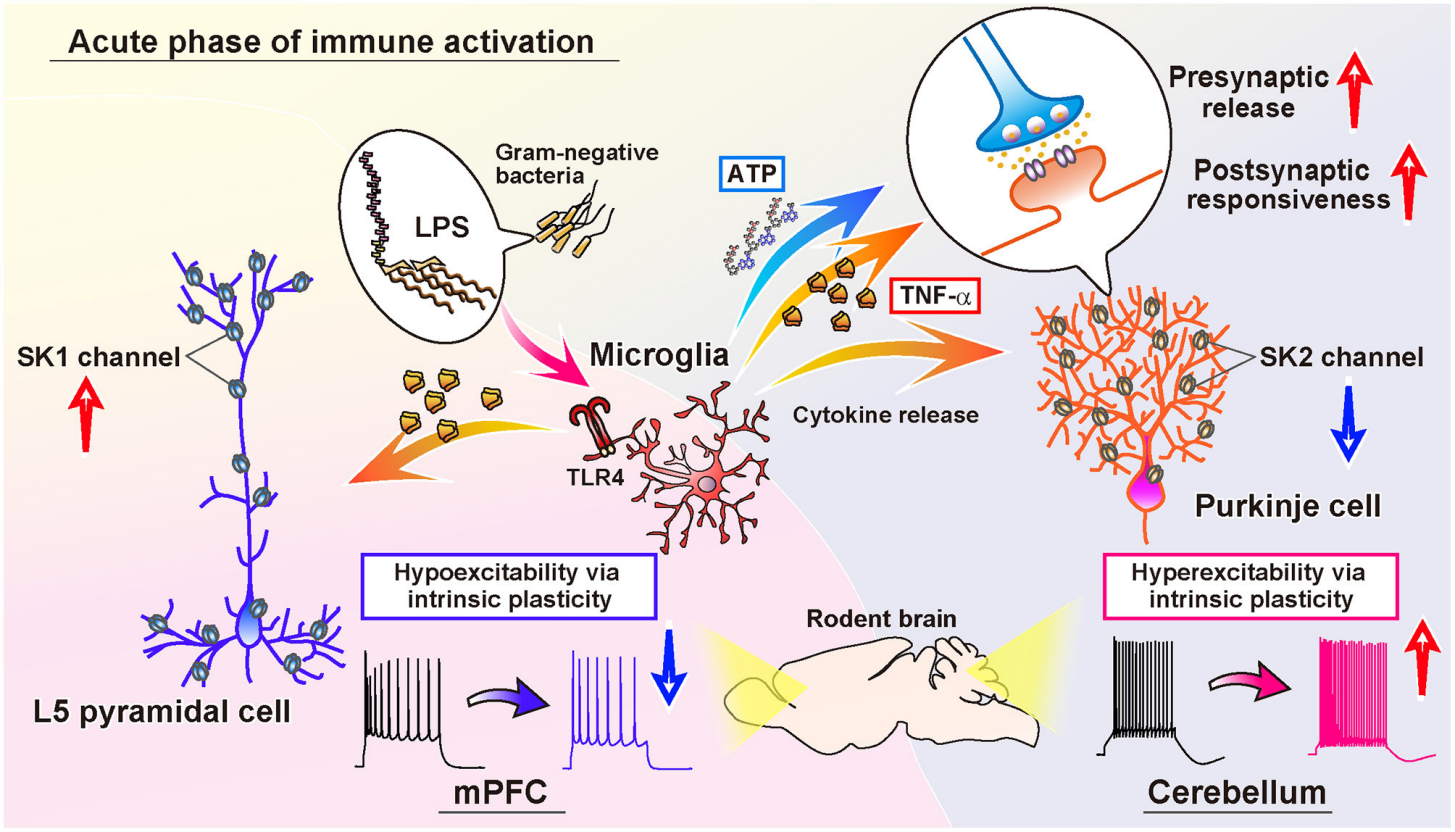
Comparison of the immune-triggered plasticity of intrinsic excitability of cerebellar Purkinje cells and mPFC L5 pyramidal cells. Exposure to LPS activates microglia in both the cerebellum and mPFC. In the cerebellum, activated microglia release inflammatory cytokines, including TNF-α, which induce downregulation of SK2 channels in the Purkinje cells. Activated microglia also contribute to ATP release in the cerebellar cortex, and both ATP and TNF-α promote the release of presynaptic vesicles of the glutamatergic terminals. Moreover, the postsynaptic responsiveness is also increased *via* intraneuronal TNF-signaling. As a result, both the amplitude and frequency of both spontaneous and miniature EPSC increases (Yamamoto et al., 2019). In the mPFC, TNF-α released from activated microglia causes the upregulation of SK1 channels of L5 pyramidal cells, in contrast to the cerebellum. Subsequently, the intrinsic excitability of L5 pyramidal cells is reduced long-lastingly. In addition, the frequency of spontaneous IPSC decreases, whereas the spontaneous EPSC does not change (Yamawaki et al., 2022).

In the cerebellar cortex, a study indicated that activated microglia by LPS-exposure induce long-term potentiation (LTP) of intrinsic excitability, as the increase in the firing frequency of action potentials, and facilitate synaptic transmission in Purkinje neurons of juvenile rodents (Yamamoto et al., 2019; Figure 1). The resultant hyperexcitability in the cerebellar cortex by acute inflammation causes an abnormality in psychomotor behaviors of rodents *via* the pathways to the cerebello-frontal cortex (Yamamoto et al., 2019; Ohtsuki et al., 2020; Ozaki et al., 2021). Depletion of microglia made the animals resistant to the LPS-exposure, and immune-triggered hyperexcitability was not induced in the microglia-depleted cerebellum (Yamamoto et al., 2019). After exposure to the endotoxin, microglia secrete tumor necrosis factor (TNF)-α rapidly through a protein-synthesis-independent manner and a non-constitutive pathway. TLR4 on the surface of microglia recognizes the endotoxin, primary response gene 88 (MyD88), and Toll/IL-1 receptor (TIR) domain-containing adaptor-inducing interferon-β (TRIF) play the downstream pathway (Yamamoto et al., 2019). The released mediator TNF-α increases in action potential firing in a translation-independent manner. Induction of intrinsic plasticity, as LTP of the intrinsic excitability of Purkinje cells, was humbled by administration of LPS, heat-killed Gram-negative bacteria, and TNF-α in advance, suggesting crosstalk of the induction mechanism between naïve form of intrinsic plasticity and immune-triggered plasticity. Pharmacological suppression of the cerebellar astrocytes, such as Bergmann glia, did not diminish from inducing hyperexcitability plasticity of Purkinje neurons (Yamamoto et al., 2019). After activating TNF receptors expressing on the neurons, activation of the protein phosphatases elicits the trafficking of ion channels. Cerebellar microglia induce phosphatase activation and downregulation of small-conductance Ca^2+^-activated K^+^ channels (SK channels), which intensifies the intrinsic excitability of Purkinje cells (Yamamoto et al., 2019; Figure 1). In Purkinje cells, SK2 channels are specifically expressed, which is also shown to augment the excitability of dendrites (Belmeguenai et al., 2010; Ohtsuki et al., 2012; Ohtsuki and Hansel, 2018; Yamamoto et al., 2019; Ohtsuki, 2020). While TNF-α does not solely target the neuronal membrane, secreted TNF-α also modulates presynaptic transmission *via* TNF receptors on astrocytes (Santello et al., 2011; Habbas et al., 2015). Another study showed that the interference of TNF receptor 1 in Bergmann glia suppressed the effect of the TNF-α on the excitability increase of Purkinje cells (Shim et al., 2018). In response to endotoxin, activated microglia lead to activating Bergmann glia. Through the activation, Bergmann glia may promote presynaptic release from excitatory parallel fibers and climbing fibers following TNF-α stimulation and ATP release (Yamamoto et al., 2019). Indeed, Yamamoto et al. (2019) showed an increase in both spontaneous excitatory postsynaptic current (EPSC) and miniature EPSC frequency, and their amplitude, suggesting the modulation of both an increase in the activity of presynaptic neurons or the local excitability of terminals (e.g., granule cells and climbing fibers), vesicular release from pre-synapses, and post-synaptic responsiveness (Ohtsuki and Hirano, 2008; Yamamoto et al., 2019). However, the machinery for synaptic plasticity is not completely characterized yet.

It is noteworthy that, generally, in synapses, there are two sites for modulation: pre- and post-synapses. The modulation of presynaptic release is identified by the increase and decrease in the frequency of miniature EPSC and inhibitory postsynaptic current (IPSC) of the excitatory and inhibitory synapses, respectively. Miniature EPSC and IPSC are the synaptic events recorded under voltage-clamp in the presence of a blocker of voltage-sensitive Na^+^-channels (i.e., tetrodotoxin, TTX). In contrast, spontaneous events are recorded without TTX. When the amplitude of miniature EPSC and IPSC changes, it indicates changes in postsynaptic responsiveness.

## Immune-Triggered Plasticity in the mPFC

In contrast to the immune-triggered plasticity in the cerebellum, transient LPS-exposure to the medial prefrontal cortex (mPFC) and the microglia activation reduces the intrinsic excitability of layer 5 (L5) and L2/3 pyramidal neurons (Yamawaki et al., 2022; Figure 1). Interestingly, the exposure to LPS did not decrease the firing frequency of fast-spiking interneurons, indicating cell-type specificity of the LPS-triggered plasticity. Such hypoexcitability plasticity—a sustained reduction of the intrinsic excitability of neurons—was prevented under the suppression of microglial activity, too. The hypoexcitability plasticity of pyramidal cells by LPS-exposure was suppressed in the preparations pre-incubated by colony-stimulating factor 1 receptor [CSF1R, also known as macrophage colony-stimulating factor receptor (M-CSFR), which is required for the maintenance of microglia (Yamawaki et al., 2022)]. Whereas the excitability of cerebellar Purkinje cells increases *via* downregulation of SK channels (Yamamoto et al., 2019), interestingly, the excitability of mPFC pyramidal neurons decreases *via* upregulation of SK1 channels in response to microglia activation and the release of inflammatory cytokine TNF-α (Yamawaki et al., 2022). The TNF-signaling to the intracellular cascade of pyramidal neurons is suggested *via* the activation of protein phosphatases in the mPFC L5 pyramidal neurons (Beattie et al., 2002; Santello et al., 2011; Pribiag and Stellwagen, 2013; Habbas et al., 2015; Yamawaki et al., 2022). This prominent comparison of the directionality of modulation of neurophysiological properties represents the inverted form of the plasticity in both cerebellar Purkinje cells and mPFC pyramidal cells. The inverted directionality of immune-triggered intrinsic excitability plasticity would be derived from both neuronal and microglial mechanisms (Yamawaki et al., 2022; Figure 1). In addition, exposure to LPS or interleukin-1β (IL-1β) did not change the spontaneous EPSC in the mPFC, whereas it decreased the frequency of spontaneous IPSC but not miniature IPSC, implying the reduction of the network activity through hypoexcitability of pyramidal cells after acute inflammation in the mPFC (Yamawaki et al., 2022). In the neocortical L5 pyramidal neurons, IFN-γ was also reported to increase amplitudes of spontaneous and miniature IPSCs without changes in frequency, suggesting postsynaptic modulation of γ-aminobutyric acid (GABA)-ergic transmission (Janach et al., 2020). Besides, IFN-γ diminished Ih current, suggesting involvement of protein kinase C (PKC), while IFN-γ did neither alter subthreshold nor suprathreshold neuronal excitability (Janach et al., 2020). Therefore, among the phases of inflammation in the CNS, the neuromodulatory effects are various, and it is also dependent on the brain region, cell type, inflammatory mediator, and related immune cells.

## Synaptic Modulation in the Spinal Cord During Inflammation

As the phenomenon of synaptic modulation during inflammation, the neural pain accompanied by hypersensitivity of neurons in the spinal cord is relatively well-studied, and it would be a good example to introduce here. In the early studies in the spinal cord, it has been shown that brain-derived neurotrophic factor (BDNF) released from microglia induces depolarization of anion reversal potential and modulates the driving force of inhibitory GABAergic channels *via* downregulation of the potassium-chloride cotransporter KCC2 (also known as: potassium-chloride transporter member 5 and SLC12A5) mediated by TrkB signaling, associated with the neural pain (Coull et al., 2003, 2005). Thus, inflammatory mediators released from immune cells, like microglia and T-cells, have various influences on the synaptic modulations depending on the type of synapses or their receptors associated with nociception and the hypersensitivity of neurons (Grace et al., 2014). In the excitatory presynaptic terminals of the spinal cord, TNF-α, IL-1β, CCL2/MCP-1, reactive oxygen species (ROS), and IFN γ increase glutamate release *via* transient receptor potential vanilloid 1 (TRPV1), transient receptor potential cation channel subfamily A member 1 (TRPA1), interleukin-1 receptor 1(IL-1R1), and presynaptic ionotropic glutamate receptor [N-methyl-D-aspartate receptor (NMDA receptor)] (Kawasaki et al., 2008; Medvedeva et al., 2008; Park et al., 2011a,b; Nishio et al., 2013; Ma et al., 2020; Donnelly et al., 2021). On the other hand, GABAergic synaptic transmission of the inhibitory interneurons is attenuated by TNF-α, IL-1β, IL-6, the pair of CLL2 and CCL2/MCP-1, and ROS (Gosselin et al., 2005; Vikman et al., 2007; Kawasaki et al., 2008; Zhang et al., 2010). CCR2 (C-C Motif Chemokine Receptor 2) is the receptor of CCL2 expressed in the neurons of brain-wide regions including the telencephalon, diencephalon, mesencephalon, cerebellum (cortex and nuclei), and brainstem (Banisadr et al., 2005a,b). Although IFN-γ was also implied to modulate the release of GABAergic transmission in the dorsal horn neurons (Vikman et al., 2007; Grace et al., 2014), bicuculline, which was used in the study, is not specific to GABA_A_ receptor but it also affects small-conductance Ca^2+^-activated K^+^ channels (Khawaled et al., 1999), and thus further investigation would be required. In general, it is required to consider that many experiments using bicuculline for specifying excitatory synaptic transmission should modulate the neuronal excitability *per se* and do not necessarily exclude the effect of excitability modulation (Takeuchi et al., 2008). As mentioned, according to Janach et al. (2020), IFN-γ receptors express in the neocortical pyramidal neurons, and the administration of IFN-γ increased both amplitudes of spontaneous and miniature IPSCs without changes in their frequency, suggesting postsynaptic modulation of GABAergic transmission (Janach et al., 2020). In the spinal cord, again, BDNF from microglia changes the driving force of GABAergic conductance *via* downregulation of the potassium-chloride cotransporter KCC2 mediated by TrkB signaling (Coull et al., 2003, 2005). Glycinergic neurotransmission is also inhibited by IL-1β and IL-6 (Kawasaki et al., 2008). As for the postsynaptic responsiveness, various inflammatory cytokines, including TNF-α, IL-1β, IL-17, ROS, CXCL1, and CXCR2 may promote phosphorylation of NMDA receptors, potentiate the efficacy of NMDA receptors, and may maintain the neuropathic pain and mechanical allodynia (Gao et al., 2007; Kleibeuker et al., 2008; Meng et al., 2013; Zhang et al., 2013). However, we have to note again that the protein expression or quantification by merely western blotting and immunostaining does not always indicate modulation of synapse functions and localization. Therefore, we consider that current NMDA receptor trafficking in the spinal cord is based on indirect evidence yet without tests with the physiological approaches. Therefore, we just describe the possibility of the functional modulation of NMDA receptors and stress the necessity of further investigation regarding immune-triggered plasticity. Katsura et al. (2006) suggested that activation of Src-family tyrosine kinases in spinal cord microglia is crucial for mechanical hypersensitivity after peripheral nerve injury, but not in neurons or astrocytes. Their results indicate no change in phosphorylation of Src-family kinase in primary sensory neurons but OX42-positive microglia. Src-family kinase in microglia contributes to mechanical hypersensitivity presumably *via* the expression of TRPV1 and TRPA1 in the sensory neurons (Katsura et al., 2006). Prostaglandins (PGs) also have a crucial role in pain sensitization (Ahmadi et al., 2002; Harvey et al., 2004), but we will discuss this physiologically active lipid in the latter section. In sum, the modulations of synaptic transmission and intrinsic excitability in the spinal cord have been gradually revealed, and inflammation certainly links to the neuronal modulations in the spinal cord associated with the sense of pain. These forms of plasticity of neuronal activity by immunity would no wonder exist in CNS regions ubiquitously (Tables 1, 2).

## Immune-Triggered Modulation of Neuronal Activity Across the Brain

The modulation of synaptic transmission and intrinsic excitability by immunity is expected to exist in CNS regions other than the cerebellum and cerebral cortex. Here, we show summary tables of the immune-triggered modulation of the synaptic efficacy and intrinsic excitability of neurons in different brain regions (Tables 1, 2). Table 1 shows our summary of the immune-triggered synaptic plasticity. We categorized studies by animal species, brain region, neuron type, synapse types, pre-/post- synaptic location, a form of plasticity (i.e., LTP and LTD), inflammatory mediators, immune-cell or glia type, a stimulant for the immune cell, and comments regarding the induction. Arbitrarily, we allowed classifying LTP or LTD if the changes last more than 20 min after administration of immune stimulant or induction molecule. We also made a summary table of the immune-triggered modulation of the intrinsic excitability (Table 2). In tables, we included astrocytes and pericytes studies to compare the microglia because the classification of the “brain immune cells” is now not simple: e.g., astrocytes also express the immunological molecules, like interleukins, which have been believed to express only in immune cells. As in these tables, immune-triggered plasticity is induced in various regions not only the cerebellum and cerebral cortex but hippocampus, amygdala, basal ganglia, ventral periaqueductal gray (PAG), and spinal cord. Now, it is no doubt to occur ubiquitous modulations of the synaptic efficacy and the intrinsic excitability of neurons by immune cells mainly *via* innate immune responses. The related inflammatory cytokines and the mechanism for induction have numerous variations. In the following sections, we discuss the roles of inflammatory mediators, cytokines, in synaptic and non-synaptic plasticity (Roles of Inflammatory Cytokines and Lipid Mediators in Synaptic and Non-synaptic Plasticity). And we describe modulations by chemokines (Modulation of Neuronal Activity by Chemokines). Lastly, we discuss the possible involvement of the immune-triggered plasticity in psychiatric diseases (Plasticity Triggered by Inflammatory Cytokines and Relevance to Psychiatric Diseases).

## Roles of Inflammatory Cytokines and Lipid Mediators in Synaptic and Non-Synaptic Plasticity

### TNF

Tumor necrosis factor (TNF) was identified in 1975 as a factor causing hemorrhagic necrosis in tumors planted in mice (Carswell et al., 1975), now it is known as a multifunctional cytokine. The TNF family has three members: TNF-α, TNF-β/LT-α, and LT-β. TNF-α is a cytokine produced by an antigen-presenting cell (APC), and two isoforms can exist: 26-kDa membrane-tethered form (mTNF-α) and 16-kDa soluble form (sTNF-α; Kriegler et al., 1988). The mTNF-α is cleaved by the inducible TNF converting enzyme (TACE) to release the form of sTNF-α (Black et al., 1997; Moss et al., 1997), which exerts its effect through TNF receptors. In the hippocampus, TNF-α, but not IL-6 and IL-10, increases the surface expression of an α-amino-3-hydroxy-5-methyl-4-isoxazolepropionic acid (AMPA) receptor subunit GluR1 and reduces the surface expression of GABA_A_ receptors (Beattie et al., 2002; Stellwagen et al., 2005). TNF-α signaling is not required for the induction of CA1 hippocampal synaptic LTP or LTD (Stellwagen and Malenka, 2006). TNF-α activates phosphatase signaling *via* TNF receptor 1 (Pribiag and Stellwagen, 2013). As per the previous sections, TNF-α released by microglia induces plasticity in the CNS. In the cerebellar Purkinje cells, transient administration of TNF-α increases the excitatory synaptic transmission and the intrinsic excitability (Yamamoto et al., 2019). In the cerebral pyramidal cells, TNF-α decreases the intrinsic excitability, and both TNF-α and IL-1β did not alter the excitatory synaptic transmission (Yamawaki et al., 2022). In the ventral PAG, using female tyrosine hydroxylase-eGFP reporter mice, it was suggested that exposure to TNF-α decreased the excitability of dopaminergic neurons along with a modest reduction in glutamatergic synaptic transmission (Pati and Kash, 2021).

### Interleukins

Activated microglia and other immune cells release not only TNF-α but also multiple types of interleukins. In this section, we discuss the roles of interleukins in plasticity induction. Interleukins are secreted in various brain regions, including the cerebellum, brainstem, hippocampus, and cerebrum, after injection of LPS to both the peripheral and central nervous systems (Quan et al., 1994). Here, we introduce the roles of IL-1, 6, and 17.

#### IL-1

IL-1 is a protein with a molecular weight of 17 kDa, which is secreted from various cells in the body other than macrophages. Its function is not only for immune cells but also for connective tissue and CNS neurons. There are two types of molecular species, IL-1α and 1β. Another molecule with homology is the IL-1 receptor antagonist (IL-1Ra), which binds to the same receptor but does not convey a signal. IL-1 signal induces the expression of IL-8 and IL-6 by activating NFκB and AP-1 *via* MyD88. There are two types of their receptors: type IR transmits a signal, but type IIR has a deficit in the cytoplasmic region and does not transmit the signal. Similar signaling is found in IL-18R and TLR family molecules (Dinarello, 2018; Fields et al., 2019).

Among interleukins, IL-1β and IL-6 had been reported to prevent the induction of synaptic and non-synaptic plasticity in the hippocampal CA1 regions couple of decades ago (Bellinger et al., 1993; Li et al., 1997; Ross et al., 2003). These studies showed the impairment or the reduction of LTP of the excitatory synaptic transmission and the population spikes by administrating interleukins before applying the tetanus stimulation (i.e., LTP induction protocol) in the hippocampal slice preparations. *In vivo*, administration of LPS to the fourth ventricle impaired hippocampal memory tasks (Min et al., 2009). In hippocampal slices, it is reported that LTP induction was facilitated following 30 ng/ml IL-1β perfusion for 10 min, and LTD induction was prevented (Nisticò et al., 2013). In mPFC pyramidal neurons, IL-1β does not change the intrinsic excitability of action potential firing (Yamawaki et al., 2022). However, there is a difference in the modulation of synaptic transmission in primary culture. Yang et al. (2005) showed that more than 10 ng/ml IL-1β decreased the frequencies but not the amplitudes of spontaneous and miniature EPSC of neurons, indicating the suppression of presynaptic release. In the same study, IL-1β increased current mediated by both NMDA receptors and L-type Ca^2+^ channels (Yang et al., 2005). In the primary culture of hippocampal neurons, it was shown that IL-1β increases NMDA receptor function and Ca^2+^ influx through activation of Src-family tyrosine kinases and subsequent phosphorylation of NR2 subunits (Viviani et al., 2003). IL-1β also reduces the level of newly synthesized proteins in dendrites following chemically induced LTP (cLTP) by inhibiting Akt/mTOR signal transduction (Prieto et al., 2019a). Another inflammatory cytokine, IL-18, on the other hand, does not show a reduction of cLTP (Prieto et al., 2019b). Together, these studies suggest that IL-1β potentially modulates various forms of neuronal activity of hippocampal neurons: synaptic activity, Ca^2+^ signaling, and mTOR signaling required for spine stabilization and memory consolidation. Nevertheless, cultured neurons are immature and without an immunological milieu, so we cannot always consider the findings as to the biological representatives of cortical responses.

In the cerebellum, iontophoretic administration of IL-1β to the cerebellum increases the firing frequency of Purkinje cells *in vivo* (Motoki et al., 2009). In an EAE model, results suggest that the waveform of spontaneous EPSC was expanded by the activity of Bergman glia, EAAT, and IL-1β, but not by TNF-α, and such modulation was reverted by administration of IL-1Ra (Mandolesi et al., 2013).

#### IL-6

A recent study by Willis et al. (2020) revealed repopulating microglia after traumatic brain injury promote brain repair in an IL-6-dependent manner (Willis et al., 2020). In general, IL-6 receptors (also known as CD126) include the membrane-bound IL-6 receptor (IL-6R) as well as the secretory soluble IL-6 receptor (sIL-6R) in the serum (Kishimoto, 2010). The secretory type has a structure in which the intracellular and transmembrane regions of the membrane-bound receptor are truncated, and the secretory receptor also exhibits IL-6 affinity similar to that of the membrane-bound receptor. IL-6R cannot function by itself because it does not have the ability for signal transduction. The IL-6R can transmit signals only by associating with gp130. gp130 is a protein widely expressed in various types of cells, and sIL-6R associated with IL-6 acquires reactivity by interacting with gp130. When the ligand binds to the IL-6R, the IL-6R associated with gp130 transmits a signal into the cell, mainly *via* two downward signaling pathways. One is the JAK-STAT pathway, in which activated JAK1/2 phosphorylates a tyrosine residue of gp130. The phosphorylated tyrosine of gp130 binds to the SH2 domain of the STAT1/3 molecule. When the transcription factor STAT1/3 is activated *via* the SH2 domain, the complex translocates into the nucleus, and binds to DNA sequences, inducing transcription of the target genes. The other is the MAPK pathway, in which Shp2 binds to gp130, it converts Ras, a small G protein, into an active form *via* the adapter protein Grb2. Then, Ras activates Raf-1, Raf-1 phosphorylates MEK, and MEK activates ERK, in turn. Activated ERK regulates gene expression after translocating into the nucleus. Surprisingly, IL-6 may alter the immune milieu of the brain parenchyma *via* peripheral stimulation. Exposure to chronic social stress deteriorates BBB integrity *via* loss of tight junction, promoting peripheral IL-6 passage across the BBB and depression behavior (Menard et al., 2017). In the rat slice preparations of the temporal cortex, IL-6 selectively reduced the amplitude of inhibitory postsynaptic currents without affecting excitatory postsynaptic currents (Garcia-Oscos et al., 2012). The IL-6-induced decrease in inhibitory postsynaptic currents is mediated by gp130 and *via* internalization of GABA_A_ receptors. IL-6 shifts the balance between synaptic inhibition and excitation to increased excitability (Garcia-Oscos et al., 2012).

#### Cerebellar IL-6

Since the discovery of IL-6 in 1986 (Kishimoto, 2010), the localization of the mRNA of IL-6 and IL-6A has been revealed in various regions of the rodent brain, including the cerebellum, medulla, neocortex, and hippocampus (Schöbitz et al., 1993; Gadient and Otten, 1994a,b). In the cerebellum, the IL-6R and gp130 express in neurons, including Purkinje cells and granule cells (Morikawa et al., 2000). And chronic administration of interleukins is neurotoxic and induces apoptosis of granule cells and Purkinje cells (Peng et al., 2005; Kaur et al., 2014). In the GFAP-IL6 transgenic mouse, IL6 is overexpressed in the brain under the control of the GFAP promoter, as a model of chronic brain inflammation (Campbell et al., 1993). According to Gyengesi et al. (2019), the motor performance of GFAP-IL6 mice showed an age-dependent difference: reduced performance on the rotarod, higher ataxia scores, and deficits in beam walking, later in life. GFAP-IL6 mice also showed an increased number of Iba1-positive microglia and high TNF-α levels, as well as a reduction of cerebellar volume, which starts at 6 months to a loss of about 50%. While synaptophysin levels as a presynaptic marker remained unchanged, PSD95 levels as a postsynaptic marker decreased in the aging GFAP-IL6 mice (Gyengesi et al., 2019). Purkinje cells of GFAP-IL6 mice show a relatively low firing rate of simple spikes, compared to wild-type. And characteristically, a significantly greater proportion of Purkinje neurons from GFAP-IL6 mice exhibited an oscillatory pattern of spontaneous firing (Nelson et al., 1999). The pause period after stimulation of a climbing fiber afferent (complex spike) of Purkinje neurons was significantly longer than control, indicating that IL-6 could act directly on Purkinje neurons to alter their physiological properties (Nelson et al., 1999). Purkinje cells that are chronically exposed to IL-6 also show elevated resting levels of intracellular calcium, an increase in both AMPA-induced and metabotropic glutamate receptor (mGluR)-activated intracellular Ca^2+^ signals, and release from intracellular Ca^2+^ stores (Nelson et al., 2002, 2004; Gruol, 2013). These studies imply the possible modulation or even disruption of the induction of various forms of plasticity of Purkinje cells (Ohtsuki and Hirano, 2008; Ohtsuki et al., 2009, 2020). Another study in cultured cerebellar granule neurons suggests chronic incubation with IL-6 at a concentration of 5 ng/ml alters NMDA receptor-mediated membrane responses, increases Ca^2+^ influx through the L-, N-, and P/Q-type voltage-gated calcium channels, and enhances neurotoxicity in a developmentally regulated manner (Qiu et al., 1995, 1998). In contrast, the following study suggests a reduction of the activity of L-type calcium channels by pretreatment of granule cell culture with 120 ng/ml IL-6 (Ma et al., 2012). Thus, we notice that modulation of the excitability of CNS neurons by inflammatory cytokines is dependent on the concentration. Rather, an increase in the levels of IL-6 and its receptors in the cerebellar cortex may correlate with the disruption of the cerebellar functions associated with the cerebellum-related psychiatric disease models.

#### IL-17

IL-17A is a glycoprotein with 21 kDa molecular weight, which is secreted as a disulfide-linked homodimer. In 1993, Rouvier et al. cloned IL-17A from the cDNA library of a murine cytotoxic T lymphocyte hybridoma (Rouvier et al., 1993; Iwakura et al., 2011; McGeachy et al., 2019). Murine IL-17A is composed of 147 amino acid residues that share 63% amino acid sequence homology with human IL-17A of 155 amino acids (Iwakura et al., 2011). Five additional cytokines, IL-17B, IL-17C, IL-17D, IL-17E (also known as IL-25), and IL-17F, were identified, and they form the IL-17 family (Kolls and Lindén, 2004). IL-17 strongly induces inflammation by interacting with a wide range of cell types (epithelial cells, endothelial cells, fibroblasts, osteoblasts, macrophages, dendritic cells, etc.), by producing other cytokines (G-CSF, GM-CSF, IL-6, IL-1β, TGFβ, TNF-α, etc.), chemokines (CCL2/MCP-1, CXCL1/GROα, CXCL2/GROβ, CXCL5, CXCL8/IL-8, CXCL10, etc.) and prostaglandins (PGE2, etc.), and by driving migration of neutrophils and macrophages. Although IL-17A and IL-17F share the highest amino acid sequence homology and perform distinct functions, the functions of IL-17B, IL-17C, and IL-17D remain less understood. IL-17A is the signature cytokine of the recently identified T helper 17 (Th17) cell subset (Iwakura et al., 2011; McGeachy et al., 2019). The IL-17 receptor (IL-17R) family includes five members (IL-17RA to IL-17RE), containing conserved extracellular fibronectin-like domain, cytoplasmic SEF/IL-17R (SEFIR) domain, and cytoplasmic C/EBPβ activation domains (Iwakura et al., 2011; McGeachy et al., 2019). Both homo- and heterodimers are functional receptors for IL-17 family cytokines. IL-17A and IL-17F activate NF-κB, MAPK, and C/EBP after binding to the receptors (Iwakura et al., 2011; McGeachy et al., 2019).

According to Ribeiro et al. (2019), the extent of the hippocampal LTP and AMPA/NMDA ratio was reduced in the IL-17-deficient mice after the short-term memory training (i.e., Y-maze) but not long-term training (i.e., Morris water maze). They showed that IL-17 produced from meningeal γδ T cells modulated synaptic plasticity induction and IL-17 promoted BDNF production in astrocytes using GFAP-IL17R deletion mice. Administration of IL-17 or BDNF amended the synaptic plasticity and behavior. Therefore, the IL-17 derived from a fetal-derived meningeal-resident γδ-T-cell subset was suggested to promote cognitive behavior (Ribeiro et al., 2019). In an early study of IL-17 expression by Moore et al. (2002), mRNA of IL-17B expresses in the cerebral cortex, hippocampus, cerebellum, and spinal cord of mice. In the cerebellum, the expression is prominent in the Purkinje cells, although cells in the granule cell layer and the molecular layer were not. Monoclonal anti-IL-17B histochemistry displays its expression in neurons and glial cells in the cerebellum, hippocampus, and cerebral cortex (Moore et al., 2002). IL-17A is also studied in the mouse models of neuropathic pain and multiple sclerosis (Luo et al., 2019; Di Filippo et al., 2021). Luo et al. (2019) demonstrated that IL-17A is mainly released from spinal cord astrocytes, and its receptor IL-17R exists in somatostatin-expressing interneurons. IL-17A increases EPSCs and decreases IPSCs in lamina II_o_ somatostatin-expressing neurons. Administration of 10 ng/ml IL-17A induces hyperexcitability of neurons in dorsal root ganglia. IL-17R antibody decreases the excitability of neurons. Selective knockdown of IL-17R in spinal somatostatin-expressing interneurons reduces paclitaxel-induced hypersensitivity after paclitaxel and neuropathic pain (Luo et al., 2019). According to Di Filippo et al. (2021), the IL-17A receptor (IL-17RA) is highly expressed in neurons in the hippocampal CA1, and exposure to 20 ng/ml IL-17A disrupts hippocampal LTP through the activation of IL-17RA and p38 mitogen-activated protein kinase (MAPK). IL-17A overexpression mimics the disruption of LTP as neurons in the EAE. The loss of IL-17A ameliorates EAE-related cognitive deficits (Di Filippo et al., 2021). In contrast, the roles of cerebellar IL-17 are not well-understood yet, and an advance investigation is required.

### TGFβ

TGFβ family members (: TGFβ1, TGFβ2, and TGFβ3) are synthesized by many cell types, including macrophages, as pre-pro-TGFβ (pre-propeptide), and the pre-propeptide is cleaved in the endoplasmic reticulum and secreted as an inactive dimer bound as a latent complex, tethered by the extracellular matrix. Serum proteinases and matrix metalloproteinases (MMPs) liberate the latent form, and TGFβ-binding to transforming growth factor beta receptors (TGFBRs) transduce the signaling in the SMAD-dependent and SMAD-independent manners (Spittau et al., 2020). TGFβ1 has been considered as an essential factor for development, differentiation, activation, reactivation, and homeostasis of both innate and adaptive immunity. Interestingly, TGFβ1 increases phosphorylation of cAMP response element-binding protein (CREB) and augments the maintenance of LTP (i.e., late-phase LTP; Caraci et al., 2015). Exogenous administration of both IL-10 and TGFβ1 facilitates induction of LTP in hippocampal CA1 neurons without any effect on short-term plasticity (Nenov et al., 2019). However, there is a possibility of secondary effect *via* regulating microglia functions (Spittau et al., 2020). Further, these studies lack an investigation of the membrane properties of neurons after exposure to TGFβ, like many studies on interleukins and endotoxin. As discussed in the latter paragraph: Meta-plasticity by brain immunity, membrane properties are crucial for inducing multiple forms of plasticity. Voltage- and Ca^2+^-dependent ion channels underlie the membrane properties, which determine the intrinsic excitability of the neurons, affect the Ca^2+^ influx through the voltage-gated and ligand-gated Ca^2+^ channels, and decide the consequence as the plasticity induction.

### Prostaglandins

Prostaglandins (PGs) are the physically active lipid mediators, which are produced in almost all tissues and involved in the induction of plasticity in the brain (Akaneya and Tsumoto, 2006; Le et al., 2010). In the inflammatory pain sensitization, PGE2 activates protein kinase A (PKA), specifically phosphorylates glycine receptor subtype GlyR alpha3 and reduces the glycinergic transmission by inhibiting GlyR3 (Harvey et al., 2004). PGE2 specifically decreased fast inhibitory synaptic current through glycine receptors, but not GABA_A_, AMPA, and NMDA receptors in the rodent spinal cord dorsal horn. PGE2, but not PGF2 alpha, PGD2, or PGI2, reduced inhibitory glycinergic synaptic transmission (Ahmadi et al., 2002). In microglia, phosphorylation of extracellular signal-regulated kinase 1/2 (ERK1/2) drives PGE2 release, which activates PGE2 receptor E-prostanoid 2 (EP2) of neurons in the dorsal horn. Thus, PGE2 contributes to signaling between microglia and neurons in pain maintenance after injury (Zhao et al., 2007). PGE2 signaling could also regulate membrane excitability (i.e., action potentials and back-propagating action potential-associated dendritic Ca^2+^ influx) and LTP in hippocampal perforant path-dentate gyrus synapses (Chen et al., 2002). A recent study indicated that striatal microglia modulate the excitability of neurons *via* IL-6 and PGE2 signaling and induce negative mood (Klawonn et al., 2021). Peripheral inflammation was also suggested to induce PGE2-mediated modulation of the dopaminergic circuit for motivation in the striatum (Fritz et al., 2016). Considering the highly broad modulation by PGs (Furuyashiki and Narumiya, 2011), microglia-produced PGs could influence memory, learning, mood, and behaviors. PGs have been traditionally thought to function mostly as mediators of acute inflammation, even in the nervous system. However, a recent view suggests the involvement of PGs in chronic inflammation. PGs crosstalk with cytokines and both PGs and cytokines may synergistically activate NF-κB to induce the expression of inflammation-related genes (Yao and Narumiya, 2019), and thus, may generate the heterogeneity in brain inflammation.

## Modulation of Neuronal Activity by Chemokines

Chemokines are classified into four main subfamilies: CXC, CC, CX3C, and C chemokines. Four cysteine residues are conserved in the amino acid sequence of a typical chemokine. All of these proteins bind with G protein-coupled receptors containing seven transmembrane domains called chemokine receptors: CXCRs, CCRs, CX3CR1, and XCR1. In mouse cerebellar slices, 50-nM chemokine (C-X-C motif) ligand 1 (CXCL1; also known as GROα and GRO1) suppressed the induction of the LTD of parallel fiber-Purkinje cell synapses (Giovannelli et al., 1998). CXCL1 and IL-8 as CXCL8 increased the number of spontaneous synaptic transmissions (Giovannelli et al., 1998). It is also reported that 60-nM CXCL2 (also known as GROβ and GRO2) increased the frequency of synaptic currents in rats (Ragozzino et al., 1998). In the hippocampus, CXCL10 (10 ng/ml) suppressed the extent of LTP (Vlkolinský et al., 2004). Acute application of CX3CL1 chemokines as fractalkine, which functions as an adhesion molecule, caused a sustained reduction of EPSC amplitude (Ragozzino et al., 2006). The CX3CL1-induced LTD is due to a decrease in postsynaptic responsiveness and is mediated by the CX3CL1 receptor (CX3CR1). CX3CL1-induced depression of EPSC was not observed in the absence of afferent fiber stimulation or AMPA receptor activation, respectively, indicating the requirement of sustained CX3CR1 activation with AMPA receptor activation for its development. Ca^2+^-, cAMP-, and Ser845 GluR1 phosphorylation-dependent process is likely to modulate CX3CL1-induced LTD. Therefore, different chemokines modulate the function of synapses *via* distinct cell signaling by influencing the balance between kinase and phosphatase activity (Ragozzino et al., 2006).

As stated previously, transient exposure to a chemokine CCL2/MCP-1 increases the presynaptic release of the spinal cord (Ma et al., 2020). In hippocampal CA1 pyramidal neurons, acute administration of CCL2 increased excitability and presynaptic transmission (Zhou et al., 2011; Duan et al., 2018). On the other hand, a transgenic mouse expressing CCL2 under regulation by GFAP promoter decreased the excitability of neurons and weakened plasticity extent, which implies that chronic exposure to the chemokine may change the homeostasis of the activity in the hippocampal circuit (Nelson et al., 2011; Bray et al., 2013). Nevertheless, not only the identification of leukocytes releasing those molecules in the brain but the effects on cerebellar synaptic transmission and intrinsic excitability of neurons are unsolved.

## Plasticity Triggered by Inflammatory Cytokines and Relevance to Psychiatric Diseases

### Involvement of IL-17 in the Developmental Disorders

Recently, IL-17 has been shown involved in the disruption of the cortical neural circuit and aberrant behavioral phenotypes (Choi et al., 2016; Reed et al., 2020), which attracted researchers. Viral infection during pregnancy has been considered correlated with increased frequency of autism spectrum disorder, ASD, in offspring. This observation has been modeled in rodents subjected to the maternal immune activation, MIA. The Th17 and IL-17 in mothers are involved in MIA-induced behavioral anomalies of offspring, representing an increase in the pup ultrasonic vocalization (USV) responses, a decrease in the social interaction, and an increase in the marble burying. MIA also induces an abnormal cortical layer formation, dependent on maternal IL-17 in the fetal brain. And the administration of anti-IL-17a antibody to the pregnant mother ameliorates both behavioral and histological phenotypes (Choi et al., 2016). Since some children with ASD show an improvement in their behavioral symptoms during a fever, a sign of systemic inflammation, the social behavior deficits in the offspring exposed to MIA can be temporarily rescued by the administration of LPS. This was accompanied by a reduction in neuronal activity in the primary somatosensory cortex dysgranular zone (S1DZ) and its hyperactivity in the behavioral phenotypes of the offspring exposed to MIA. It was also due to an increase in IL-17 in S1DZ (Reed et al., 2020). However, the study did not answer why the administration of LPS rescued the deficits of sociability in the MIA offspring and how neuronal hyperexcitability was attenuated. Further, such studies did not see the activity in the different regions of the MIA-offspring brain nor the brain-wide connectivity, so the effect of MIA was merely investigated in the studied regions.

### Meta-Plasticity by Brain Immunity

As seen above, the immune cell activation and inflammatory mediators modulate the intrinsic excitability of neurons *via* such as SK channels and voltage-gated Ca^2+^ channels. Modulation of the intrinsic excitability of neurons could determine the induction of synaptic plasticity. The modulation of intrinsic excitability of neurons, in turn, changes the membrane voltage of neurons, which affects the driving force of ion current through channels, and the Ca^2+^ influx *via* voltage-gated Ca^2+^ channels. The membrane properties determine the induction of synaptic plasticity. It is because the induction of activity-dependent synaptic plasticity relies on the activation of the Ca^2+^ influx and downward signaling of the kinases and phosphatases, which drive exocytosis and endocytosis of neurotransmitter channels, including AMPAR, NMDAR, mGluR, GABA_A_R, GABA_B_R, glycine receptor, and so on. Indeed, the exposure to TNF-α to cerebellar Purkinje cells increases their excitability, suppresses the induction of further intrinsic plasticity, and potentiates both pre- and post-synaptic efficacy. It could imply that further induction of plasticity at granule cell synapses is occluded since the cellular signaling of intrinsic plasticity and microglia-triggered intrinsic plasticity share the intracellular signaling (Yamamoto et al., 2019). Hyperexcitability of the cerebellar cortex after LPS injection lasts at least 6 h (Yamamoto et al., 2019) so that further induction of plasticity and learning could be hampered for the duration. In the mPFC, it is speculated that further induction of synaptic plasticity is suppressed after activation of microglia and exposure to TNF-α (Yamawaki et al., 2022). Akin to this, the concept of “meta-plasticity” as the plasticity of synaptic plasticity is coined by Drs. Wickliffe C. Abraham and Mark F. Bear (Abraham and Bear, 1996; Abraham, 2008; Hulme et al., 2013). Brain immunity should give another field of research, again. It is also elusive how spike-timing-dependent plasticity (Zhang et al., 1998; Wang et al., 2000; Dan and Poo, 2004; Jörntell and Hansel, 2006; Suvrathan et al., 2016; Sgritta et al., 2017; Ohtsuki et al., 2020) is modulated by aberrant immunity or in the animal models with brain inflammation. Chronic administration of excess immunity during development may modulate the neuronal activity long-lastingly. In dendrites, the intrinsic excitability influences the directionality of the LTP/LTD induction (Wang et al., 2003; Sjöström and Häusser, 2006; Sjöström et al., 2008) and the conductivity of synaptic transmission along dendrites (Ohtsuki et al., 2012; Ohtsuki and Hansel, 2018; Ohtsuki, 2020). Considering those, many previous studies just showing the correlation between the impairment of synaptic plasticity and disruption of memory by immune stimulants would be required reinvestigation. New findings are expected to come from such efforts. Concurrently, it would be required to prove the identification of not only inflammatory mediators but also source immune cells by abolishing related cells (by depletion of microglia and T cells pharmacologically or genetically) and by regulating the activity of specific immune cells with gene manipulation.

### Disease Association of the Immune-Triggered Neuroplasticity

Despite the difficulty in interpreting the biological significance of behaviors, learning, and cognition, the interaction of immune cells with neurons is a quite important feature of brain functioning. The immune-triggered plasticity and related meta-plasticity are expected to be involved in psychiatric disorders evoked by aberrant immunity, specifically at the stage of neurodevelopment and maturation of brain vasculatures (Hopkins and Rothwell, 1995; Gilmore et al., 2004; Choi et al., 2016; Menard et al., 2017; Greene et al., 2018; Nie et al., 2018; Segawa et al., 2021; Parvez and Ohtsuki, 2022). It is also plausible that brain immunity, such as microglia or disease-associated macrophages in the brain, may have an “immune-memory” in the ways of responses against immune stimulants *via* an inflammatory mediator signaling and plasticity. Subsequently, the myelination of neuronal axons might also become impaired, which is seen in autism patients (Ben Bashat et al., 2007) and schizophrenia model animals (Hirahara et al., 2013). Questions are the timing when brain functions are severely affected; which brain regions are vulnerable; what specificity and localization the brain immune cells have; how long immune reactivity maintains; what the molecular or genetic mechanisms in immune cells and neurons are; and whether we can suppress or revert the reactivity. In the disease brains, resultant anomalies in the brain-wide functional connectivity caused by immune-triggered plasticity have been found recently (Yamamoto et al., 2019; Tsurugizawa et al., 2020), and the pathways for diseases would be clarified (Ohtsuki et al., 2020; Wang et al., 2021). Those efforts should help us to clarify the target brain regions, immune-cell targets, or pathways for emerging the disease and to ameliorate symptoms with the drug delivery. Highly multiplexed special expression pattern of distinct marker proteins with multi-dimensional data analyses must enable us to interpret the whole embodied schema of immune-triggered plasticity and disease association in the brain microenvironment and tissue architecture, as done in the field of cancer (Fernández-Zapata et al., 2020; Chen et al., 2021; van Maldegem et al., 2021; Kuett et al., 2022). Additionally, ion channels significant for the intrinsic excitability plasticity of neurons, such as Ca^2+^-activated K^+^-channels (i.e., SK-, IK-, and BK-channels) or HCN channels, would be another potential target for the drug administration, while we should note that inflammatory mediators could be modulators of not only fast synaptic transmissions and the intrinsic excitability but also metabotropic receptors of monoamine, mGluRs, GABA_B_R, and other G protein-coupled receptors, which are also involved in the plasticity induction.

## Conclusion

In this review, we introduced the induction of immune-triggered plasticity of neurons across different brain regions. Typically, in the cerebellum, the Purkinje cells showed hyperexcitability plasticity of action potential firing, as well as an increase in excitatory synaptic transmission. In contrast, the mPFC pyramidal cells showed hypoexcitability plasticity as the reduction of action potential firing (Figure 1). In other brain regions, such as the spinal cord, midbrain, hippocampus, amygdala, and basal ganglia, neurons show different forms of plasticity, and it is obvious that inflammatory mediators such as inflammatory cytokines, chemokines, and neurotrophic factors change the activity of neurons in different ways in the different brain immune milieu. Further, the exposure to inflammatory cytokines influences the induction of the following plasticity in the ways of reduction or facilitation of the induction of plasticity, as an immune-mediated meta-plasticity. However, it is more complex than easily considered because the modulation of the intrinsic excitability of neurons by transient and chronic activation of the brain immunity also alters the induction of synaptic plasticity and disease association. Therefore, this field is quite broad and full of phenomena highly of biological significance.

## Author Contributions

GO designed the research. MH, TM, TK, and GO wrote the original manuscript. YW, TS, and GO edited the manuscript. MH, TK, and GO revised the manuscript. All authors contributed to the article and approved the submitted version.

## Funding

This work was supported by grants from the Mitsubishi Foundation, the Takeda Science Foundation, and JSPS Kakenhi Grant-in-Aid for Scientific Research (B). The funders had no role in the study design, decision to publish, or preparation of the manuscript.

## Conflict of Interest

The authors declare that the research was conducted in the absence of any commercial or financial relationships that could be construed as a potential conflict of interest.

## Publisher's Note

All claims expressed in this article are solely those of the authors and do not necessarily represent those of their affiliated organizations, or those of the publisher, the editors and the reviewers. Any product that may be evaluated in this article, or claim that may be made by its manufacturer, is not guaranteed or endorsed by the publisher.
